# Association of rodent-borne *Leptospira* spp. with urban environments in Malaysian Borneo

**DOI:** 10.1371/journal.pntd.0007141

**Published:** 2019-02-27

**Authors:** Kim R. Blasdell, Serge Morand, David Perera, Cadhla Firth

**Affiliations:** 1 Health and Biosecurity Business Unit, Commonwealth Scientific and Industrial Research Organisation, Geelong, Victoria, Australia; 2 Animals, Health, Territories, Risks and Ecosystems, French Agricultural Research Centre for International Development, Montpellier, France; 3 Institut des Sciences de l’Evolution de Montpellier, National Center for Scientific Research, Montpellier University, Montpellier, France; 4 Faculty of Veterinary Technology, Kasetsart University, Bangkok, Thailand; 5 The Institute of Health and Community Medicine, Universiti Malaysia Sarawak, Kota Samarahan, Sarawak, Malaysia; 6 School of BioSciences, The University of Melbourne, Parkville, Victoria, Australia; Institut Pasteur, FRANCE

## Abstract

Although leptospirosis is traditionally considered a disease of rural, agricultural and flooded environments, *Leptospira* spp. are found in a range of habitats and infect numerous host species, with rodents among the most significant reservoirs and vectors. To explore the local ecology of *Leptospira* spp. in a city experiencing rapid urbanization, we assessed *Leptospira* prevalence in rodents from three locations in Malaysian Borneo with differing levels of anthropogenic influence: 1) high but stable influence (urban); 2) moderate yet increasing (developing); and 3) low (rural). A total of 116 urban, 122 developing and 78 rural rodents were sampled, with the majority of individuals assigned to either the *Rattus rattus* lineage R3 (n = 165) or *Sundamys muelleri* (n = 100). *Leptospira* spp. DNA was detected in 31.6% of all rodents, with more urban rodents positive (44.8%), than developing (32.0%) or rural rodents (28.1%), and these differences were statistically significant. The majority of positive samples were identified by sequence comparison to belong to known human pathogens *L*. *interrogans* (n = 57) and *L*. *borgpetersenii* (n = 38). Statistical analyses revealed that both *Leptospira* species occurred more commonly at sites with higher anthropogenic influence, particularly those with a combination of commercial and residential activity, while *L*. *interrogans* infection was also associated with low forest cover, and *L*. *borgpetersenii* was more likely to be identified at sites without natural bodies of water. This study suggests that some features associated with urbanization may promote the circulation of *Leptospira* spp., resulting in a potential public health risk in cities that may be substantially underestimated.

## Author summary

Leptospirosis is a significant zoonotic disease that is found in a range of environments worldwide, most notably tropical regions prone to flooding. The bacterial agents of this disease, *Leptospira* spp., are most often associated with rodents, including species frequently found in urban areas. In cities, rodent populations are often larger and denser than those found in natural environments, which can lead to higher rates of contact with people and impact human disease risk. To investigate the impacts of urbanization on *Leptospira* spp., we sampled rodents at locations with differing levels of human influence, from highly urbanized to rural, surrounding a city in Malaysian Borneo. We found that 31.6% of all rodents were positive for *Leptospira* spp. DNA, and that two primary species were present, *L*. *interrogans* and *L*. *borgpetersenii*, both of which are known human pathogens. Statistical analyses revealed that infected animals were more common in areas with higher levels of human influence, and were more likely to occur at sites with limited forest cover, and mixed commercial and residential activity. Our study adds to a growing body of evidence suggesting that there is a significant yet underappreciated risk of leptospirosis for people living in urban environments.

## Introduction

Leptospirosis is the most widespread zoonotic disease globally, with over a million cases of severe disease and around 60,000 deaths reported annually [1]. Occurring in a wide variety of environmental settings, and with the greatest impact on public health in tropical and subtropical regions, it is a significantly under-diagnosed disease due to its broad clinical picture and symptoms that are common to several other diseases [2]. Leptospirosis is caused by spirochaetes of the genus *Leptospira*, of which 22 species and >300 serovars are currently recognized. Ten species have been definitively associated with severe human disease, whilst a further five have been linked to milder disease [3]. In addition, 12 novel species have recently been identified from tropical soils, although none have yet been associated with disease [4].

Human infection with *Leptospira* spp. occurs via several routes, including through direct contact with urine or tissues from infected animals, or indirectly through contamination of (usually humid) environments with infected urine. The two species responsible for the majority of human infections, *L*. *interrogans* and *L*. *borgpetersenii*, differ in their transmission routes; *L*. *interrogans* remains viable for extended periods in aquatic or humid environments, whilst *L*. *borgpetersenii*, which has lost several genes related to environmental sensing, now relies primarily on direct transmission between hosts [5]. These differences impact the ability of each species to persist in the environment and have led to differences in distribution and zoonotic potential [6]. As such, whilst exposure to wetlands has traditionally been considered a significant risk factor for this disease, *Leptospira* spp. have been detected in a number of environments, including cities [7–10]. Although relatively little is known about the ecology and epidemiology of *Leptospira* spp. in urban environments, zoonotic transmission has been repeatedly documented and often associated with poor sanitation and slum conditions [11–14].

By 2050, 66% of the global human population is predicted to reside in urban environments and as such, the majority of human-wildlife interactions are likely to occur in these areas [15]. Critically, features of the urban environment can impact disease dynamics in wildlife hosts and increase the frequency of human exposure to zoonotic pathogens. Indeed, *Leptospira* spp., infection prevalence has been found to be higher in wildlife occupying urban habitats than natural environments, and this trend appears to be particularly significant for rodents [16]. Several species of rodent, including *Rattus norvegicus*, *R*. *rattus* and *R*. *exulans*, appear to benefit from urbanization and thrive in city environments, resulting in regular human exposure to these species and their excreta [17,18]. Despite the obvious risks posed by urban rodent infestation, the distribution, prevalence, diversity and dynamics of *Leptospira* spp. in urban populations remains largely unknown, impacting the ability of local authorities to develop effective prevention and control strategies.

In Southeast Asia, the number of reported cases and outbreaks of leptospirosis has increased dramatically in recent years, due in part to improvements in diagnosis and surveillance, but also as a result of the rapid environmental changes occurring in this region [19–21]. At least six zoonotic species have been detected in Southeast Asian rodents to date: *L*. *borgpetersenii*, *L*. *interrogans*, *L*. *kirschneri*, *L*. *weilli*, *L*. *noguchii* and *L*. *wolfii* [6,22]. In Malaysia, the annual number of reported cases increased more than 14-fold between 2004 and 2012, which led to the classification of leptospirosis as a mandatory notifiable disease at the end of 2010 [23]. Although many recent Malaysian outbreaks have been associated with outdoor recreational activities, human infections have also been documented in urban environments [24]. Some studies have begun to assess the prevalence of *Leptospira* spp. in urban reservoir species in Southeast Asia [22,25]), but none have yet compared how distribution and transmission varies with the degree of anthropogenic influence across an urban landscape. In this study, we screened native and invasive rodents found in urban, developing and rural locations around the city of Kuching, Sarawak for *Leptospira* spp., to begin to explore how urbanization effects the presence and prevalence of *Leptospira* in Malaysian Borneo.

## Materials and methods

### Ethics statement

This study was approved by the CSIRO Australian Animal Health Laboratory’s Animal Ethics Committee (#1750) and the Sarawak Forests Department (Permit: NCCD.907.4.4 (JLD.12)-131).

### Study locations

#### Urban location “Central Kuching”

**Fig 1:**
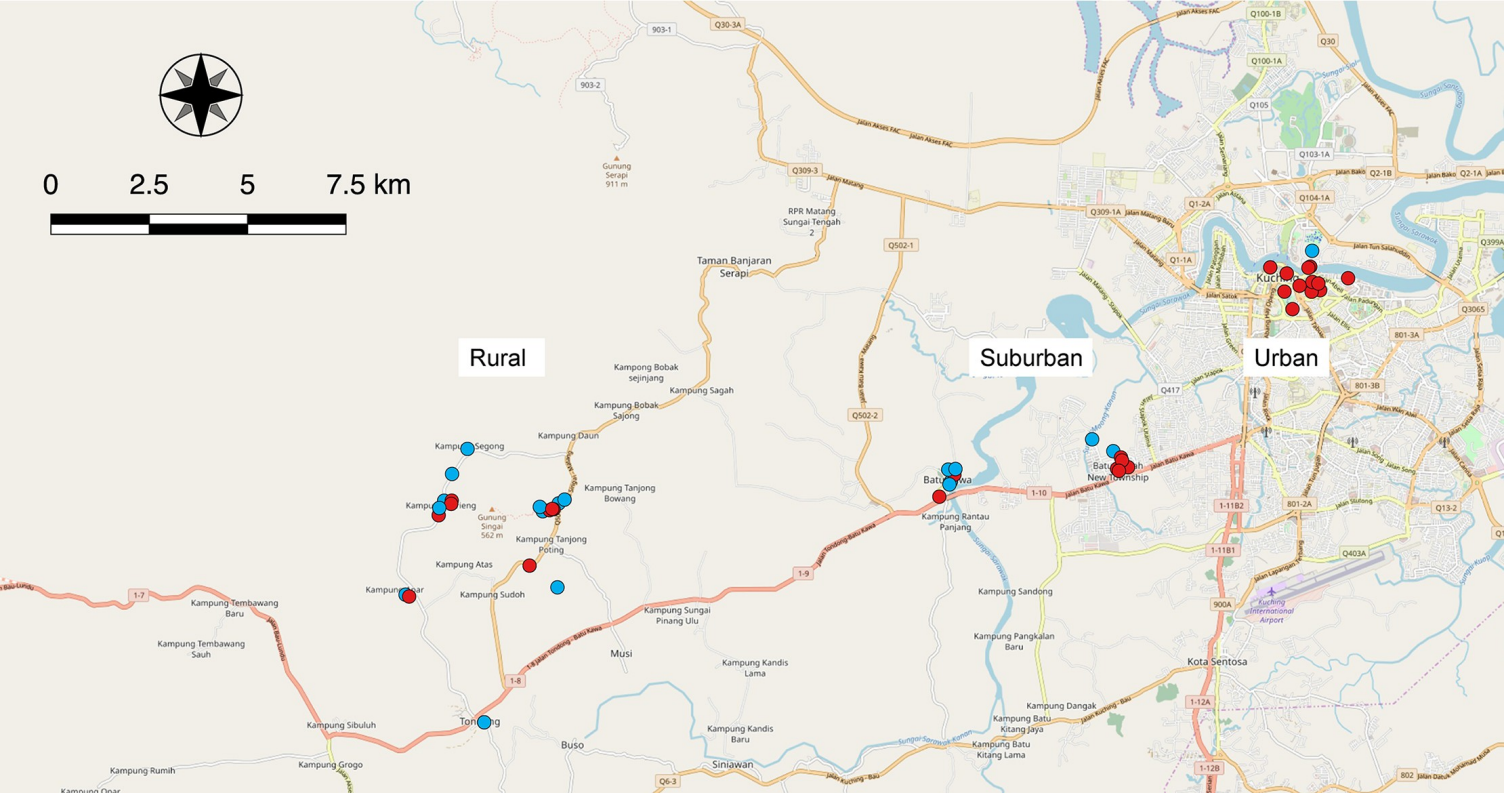
Sites of rodent collection. From left-to-right: rural (Mount Singai region), developing (Batu Kawa district), and urban (Central Kuching) locations are shown. Red dots indicate sites with rodents that were *Leptospira* spp. positive; blue dots indicate sites where no *Leptospira* spp. was detected. Map data sourced from OpenStreetMap.

Central Kuching is a highly urbanized area comprising a mix of residential and commercial buildings, interspersed with vacant lots and parks. Forest covers approximately 7% of this location and impervious surfaces dominate the landscape (see section on site environmental analysis for details). The Sarawak River flows east to west through the city, with the majority of the city to the south of the river. Due to the high anthropogenic influence in this location, the opportunities for interaction between people and rodents is high. Thirteen sites were sampled at this location, with 3 located north of the river and the remainder to the south (Fig 1).

#### Developing location “Batu Kawa district”

Batu Kawa is a rapidly urbanizing district ~8.5 km to the west of the Central Kuching location. Batu Kawa district contains a mix of residential, commercial and industrial properties, with large tracts of undeveloped, but often highly disturbed, vegetation and is approximately 39% forested. The Sarawak River runs north to south between the original township of Batu Kawa to the west and the newer developments to the east, where the intensity of development is higher (Fig 1). The anthropogenic influence at most sites in this location is high, providing ample opportunities for human-rodent interactions. Of the 14 sites sampled at this location, 9 were located to the east of the river with the remainder around the original township (Fig 1).

#### Rural location “Mount Singai region”

This rural region consists of a number of villages encircling Mount Singai, approximately 22.5km west of the Central Kuching location. The intensity of development is low, with residences consisting of a combination of traditional and modern buildings, surrounded by small adjacent rice fields and modest subsistence farms. The villages are primarily dispersed and separated by tracts of disturbed forest and scrub. Mean forest cover is highest at this location at 81%, and green space dominates the landscape. The anthropogenic influence in this location is variable, with greater opportunities for human-rodent interactions in the villages than vegetated areas, although residents frequently engage in foraging and hunting activities. A total of 19 sites were sampled at this location (Fig 1).

### Site environmental analysis

For this study, sites were considered to be a circle with a 110 m radius centered at the point where GPS coordinates were taken during rodent trapping. All site-specific environmental variables were measured or estimated over the complete circle. The 110 m radius was chosen to correspond with the approximate home range of *R*. *rattus* that has been estimated under similar environmental conditions [26]. As home range data is not available for the other rodent species studied, we used the *R*. *rattus* estimate to delineate sites throughout the study.

To classify the degree of urbanization and the intensity of anthropogenic influence at each site, the following estimates of land use were considered: 1) Mean forest cover was estimated using QGIS v 2.14.0 and previously published forest cover and loss datasets at the Landsat pixel scale. Mean estimates were ranked and grouped into tertiles, which were categorized as minimal, moderate or maximal forest cover (https://earthenginepartners.appspot.com/science-2013-global-forest) [21]. 2) Dominant land-cover type (gray, green or gray/green interface) was determined by assessing the proportion of vegetated (forest, scrub, etc.) or impervious (buildings, roads, etc.) space within and around each site using QGIS (as above) and ground-truthing. Gray sites were considered to be completely within and primarily surrounded by human infrastructure, green sites were those dominated by unmanaged vegetation, gray interface sites were within human infrastructure but adjacent to substantial vegetation, and green interface sites were within managed/unkempt vegetation and adjacent to human infrastructure. Other site-specific environmental features recorded included the presence or absence of a natural water body at a site, and the local environment in which individual rodents were caught, referred to as ‘trap location’. Trap locations were recorded as: 1) inside domestic dwellings, 2) household gardens and yards, 3) forests, 4) sewers, and 5) scrub (areas of vegetation dominated by unkempt bushes and grasses). Where buildings were present at a site, the relative condition (i.e., poor, fair, good, excellent) and type of building(s) (i.e., residential, mixed commercial/residential, institutional) were also recorded.

### Rodent sampling and speciation

Rodents were collected from multiple sites between September 2015 and April 2016 at each of the three locations described above. At each site, multiple wire mesh traps (~30cm x 14cm) were baited with meat and banana, placed at intervals >1m for between one and seven nights, and checked every morning. Trapping effort varied substantially between sites in an effort to collect equal numbers of animals/species/location. Rodents were euthanized by over-anesthetization in isoflurane, followed by bilateral thoracotomy. Sex, reproductive status, weight (as a proxy for age) and tentative species assignment (by morphological assessment) were recorded, and tissues were collected and frozen directly on dry ice. The species identity of each animal was confirmed by sequencing the product of a PCR assay using primers BatL5310 and R6036R, which amplify 726bp of the *cytochrome oxidase I* gene [27].

### Detection of *Leptospira* DNA

Approximately 30mg of rodent kidney was homogenized in 600ml of Buffer RLT Plus (Qiagen) containing 1% β-mercaptoethanol using the TissueLyser II (Qiagen), and a 5mm stainless steel bead. Homogenized tissue was clarified by centrifugation and the resultant supernatant transferred to a new tube and used for DNA extraction with the AllPrep DNA/RNA mini Kit (Qiagen), as per the manufacturer’s instructions. DNA quantity and quality were assessed using a NanoDrop (Thermo Scientific), diluted to <400ng/ul, and subjected to six previously described PCR assays targeting the *rpoB*, *flaB* and 16S rRNA genes [28–34]. Multiple PCR assays were chosen to maximize the probability of detecting any and all *Leptospira* spp. DNA present, including both pathogenic and non-pathogenic species. Samples were considered positive if they produced a visible band on an electrophoresis gel that could be confirmed as *Leptospira* spp. by Sanger sequencing (conventional PCRs), or if they demonstrated a Ct value of 35 or lower by *Leptospira*-specific TaqMan PCR. The resultant sequences (S1 Appendix) were trimmed for quality and length and subjected to BLAST (http://blast.ncbi.nlm.nih.gov/Blast.cgi) analysis to assess sequence similarity and determine putative species [3]. Sequences were considered to belong to a species if they shared ≥99% nucleotide similarity with publicly available sequences from verified species.

### Statistical analyses

Statistical analyses were conducted for all *Leptospira* spp., as well as for *L*. *interrogans* and *L*. *borgpetersenii* separately, due to documented differences in transmission routes [5]. We further considered all rodent hosts collectively to avoid conflating *Leptospira* ecology with rodent ecology, as no evidence exists at present to suggest that these rodent species differ in competence [6]. Chi squared tests were used to assess differences in *Leptospira* prevalence in rodents between all three locations (i.e., urban, developing, and rural), as well as between each pair of locations.

To interrogate the relationships between site-specific environmental variables, the GoodmanKruskal package (version 0.01) implemented in R was used to run Goodman and Kruskal’s tau (τ) statistic (https://CRAN.R-project.org/package=GoodmanKruskal) [35]. This test measures the strength of associations between categorical data, with values ranging from −1 (perfectly negative association) to +1 (perfectly positive association). A multivariate analysis of mixed data was also performed using the package PCAmixdata (version 3.1) implemented in R [36].

To examine links between the probability of *Leptospira* infection of rodents and site-specific environmental variables, a generalized linear (mixed) model (GLMM or GLM) with logit function was created using the lme4 package implemented in R (version 1.1–15) [37]. Analyses were performed using two initial models with explanatory variables chosen according to the strength of association identified in the Goodman and Kruskal’s tau statistics, and the results of the multivariate analysis. The first model (a GLMM, henceforth referred to as the global model), included the following explanatory variables: site location, trap location, forest cover, dominant land-cover type and waterbody, with no interactions added among independent variables and rodent species as a random effect. The second model (a GLM, referred to as the built environment model) was intended to investigate aspects of the built environment that may be relevant to *Leptospira* prevalence, and included site location, trap location, building type and building condition, with no interactions among them. Only the infection status of rodents trapped in sites with buildings present were included in this model. Support for competing models was evaluated using the Akaike information criterion adjusted for small sample sizes (AICc) in the package AICcmodavg (version 2.1–1), and Akaike weights w_r_ [38]. Selection of the best models was made using the R package glmulti (version 1.0.7) [39]. The three top best models selected for the global and built environment modes are given in S2 Appendix and S3 Appendix, respectively.

## Results

**Fig 2:**
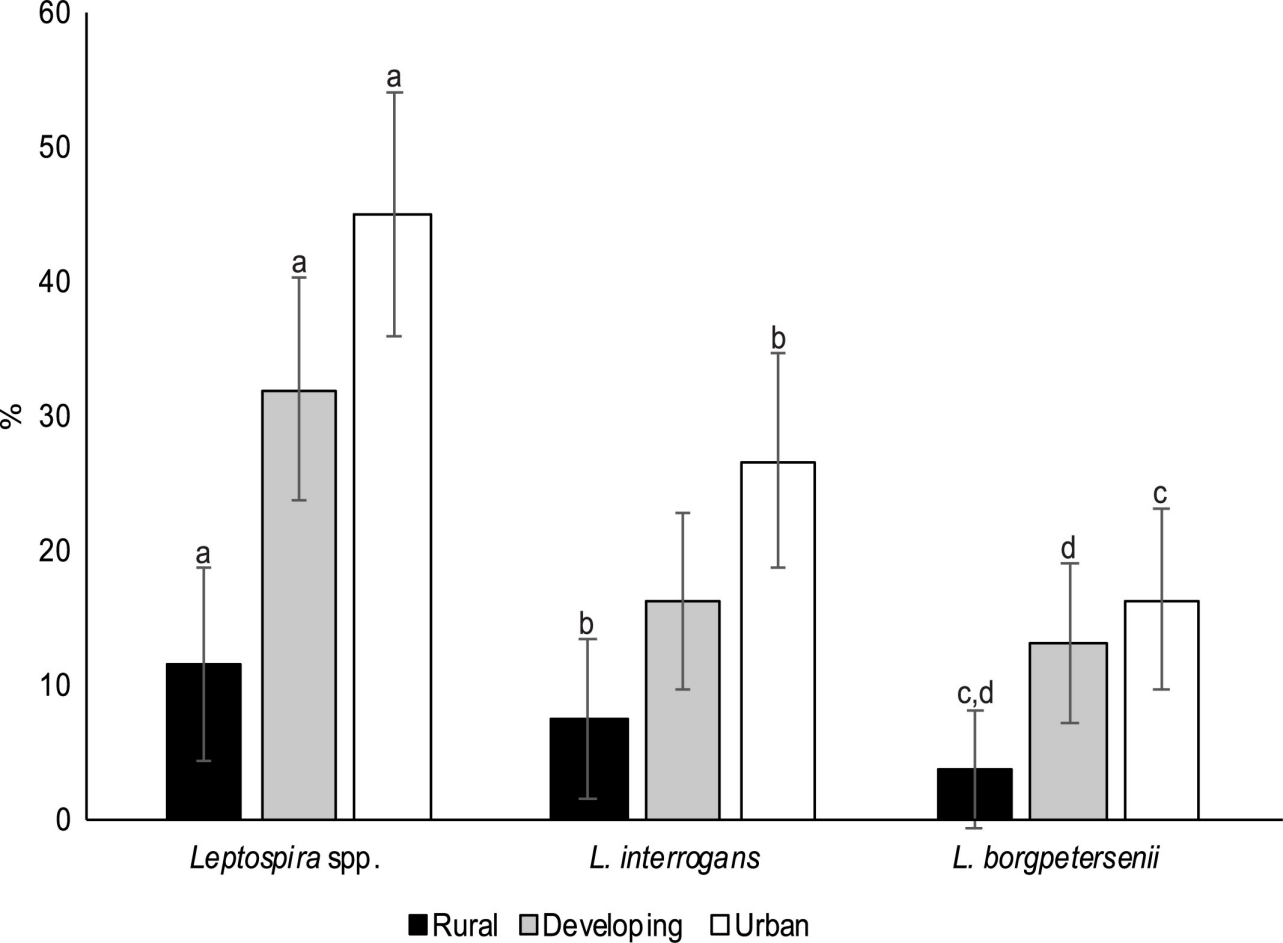
Proportion (%) of individuals positive for *L*. *interrogans*, *L*. *borgpetersenii* and all *Leptospira* by site location. Vertical lines show 95% confidence intervals, and bars labelled with the same lowercase letters are significantly different from each other.

A total of 316 animals were caught across all locations. Of these, nine species from four genera were identified by COI sequence analysis, with most individuals classified as *S*. *muelleri* (n = 100 individuals) or as *R*. *rattus* R3 (n = 165), one of the lineages within the *R*. *rattus* super-group (Table 1) [40,41]. A total of 31.6% of all animals were positive for *Leptospira* spp., and *Leptospira* spp. prevalence varied significantly by site location, with rodents from urban and developing locations more likely to be infected than rural rodents (Fig 2; S2 Appendix).

**Table 1:** Proportion of *Leptospira* spp. positive rodents by location and species.

Species	N positive/N tested (%)			Total
	Rural	Developing	Urban	
*R*. *rattus* R3*	3/30 (10.0)	29/61 (47.5)	38/74 (51.4)	70/165 (42.4)
*R*. tanezumi*	-	2/6 (33.3)	1/5 (20.0)	3/11 (27.3)
*R*. tiomanicus*	-	2/8 (25.0)	-	2/8 (12.5)
*R*. *exulans*	0/2 (0)	0/1 (0)	-	0/3 (0)
*S*. *muelleri*	4/22 (18.2)	6/41 (14.6)	13/37 (35.1)	23/100 (23.0)
*Maxomys ochraceiventer*	1/2 (50.0)	-	-	1/2 (50.0)
*M*. *whiteheadii*	1/11 (9.1)	0/1(0)	-	1/12 (8.3)
*Niviventer cremoriventer*	0/10 (0)	0/4 (0)	-	5/14 (7.1)
*N*. *sp*.	0/1 (0)	-	-	0/1 (0)
**Total**	9/78 (11.5)	39/122 (32.0)	52 /116 (44.8)	100/316 (31.6)

*Member of the *R*. *rattus* super-group

Sequence analysis revealed the presence of two distinct *Leptospira* species: *L*. *borgpetersenii* and *L*. *interrogans*. *L*. *borgpetersenii* was identified in 38 rodents (six *S*. *muelleri* and 32 *Rattus* spp.), whilst *L*. *interrogans* was identified from 57 rodents, comprising *Maxomys ochraceiventer* (N = 1), *M*. *whiteheadii* (N = 1), *S*. *muelleri* (N = 15), and *Rattus* spp. (N = 40). Sequence information could not be obtained from five samples, which were positive only by a qPCR assay that yielded amplicons too small to sequence [34].

**Fig 3:**
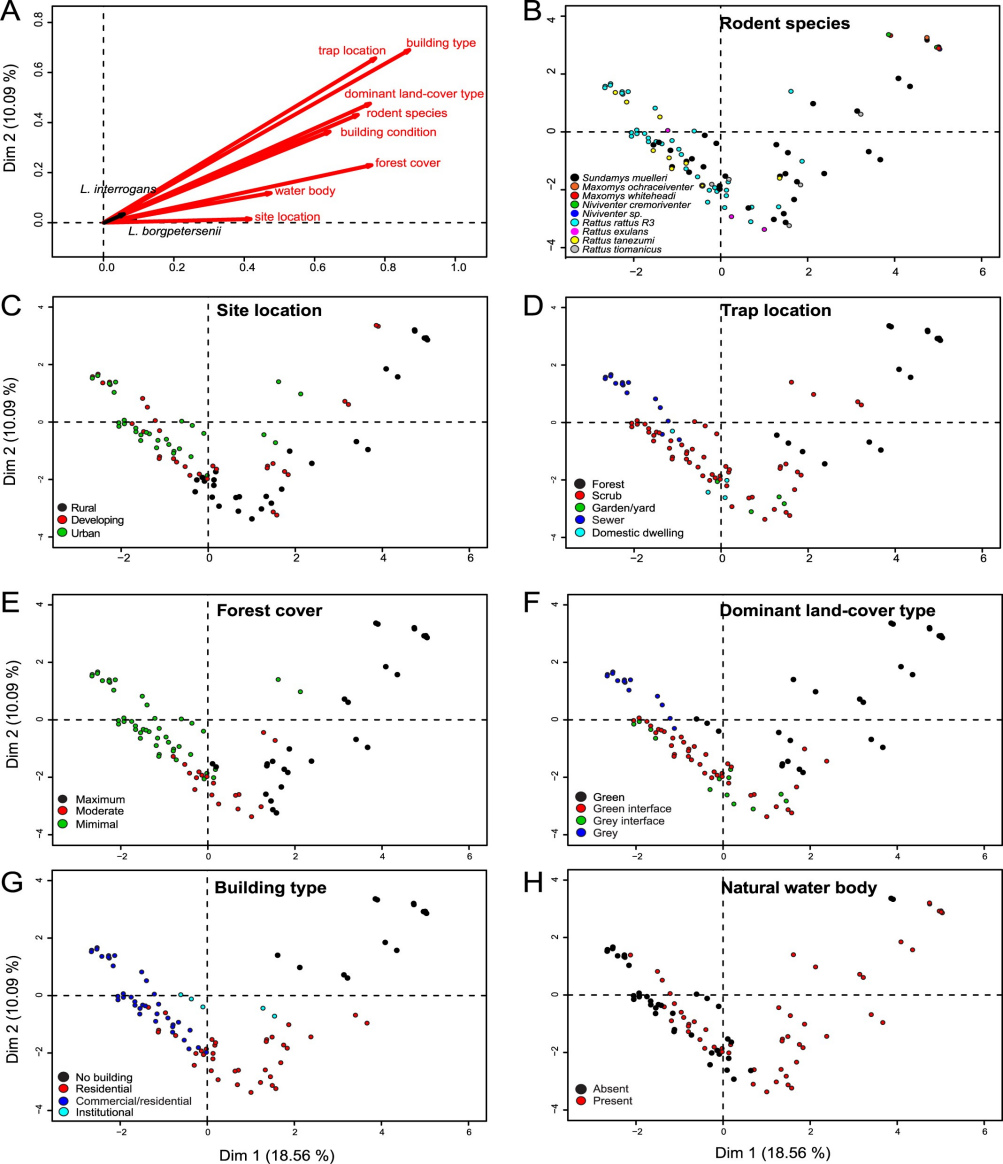
Multivariate analysis of the environmental variables included in the initial global GLMM. (A) Plot of all categorical variables; (B) plot of the species identity of individual rodents; (C-H) plot of individual rodents in relation to various environmental variables.

Goodman and Kruskal’s τ and our multivariate analysis showed moderate positive associations between pairs of environmental variables, but as all values were <0.60, they were not considered to be fully redundant in this case (S3 Appendix) (Fig 3). As a result, no variables were excluded from the subsequent analyses. Of the variables considered in the global GLMM, trap location, forest cover, dominant land-cover type and water body were each important in explaining the infection of rodents with *Leptospira* (S4 Appendix). Significant associations were identified in the first top model between infection with any *Leptospira* species and sites characterized by minimal forest cover (P = 0.001), and the absence of natural water bodies (P = 0.01), which may be reflective of the association between minimal forest cover and *L*. *interrogans*, and the absence of a natural water body with *L*. *borgpetersenii* (Table 2).

**Table 2:** Results of the first best global GLMM (with logit link function and rodent species as a random factor), explaining the occurrence of (1) all *Leptospira*, (2) *L*. *interrogans*, and (3) *L*. *borgpetersenii* in rodents as a function of environmental variables.

	Explanatory variables	Estimate (SD), P	Log likelihood, dev (DF)	AICc
All *Leptospira*	Forest cover			
	moderate *vs* maximal	0.21 (0.53), 0.70		
	minimal *vs* maximal	1.34 (0.41), **0.001**		
	Water body			
	presence vs absence	-0.83 (0.32), **0.010**	-172.1, 344.3 (311)	354.3
*L*. *interrogans*	Forest cover			
	moderate *vs* maximal	0.49 (0.74), 0.51		
	minimal *vs* maximal	1.41 (0.63), **0.027**		
	Dominant land-cover type			
	green interface *vs* green	-0.12 (0.56), 0.84		
	grey interface *vs* green	-0.35 (0.61), 0.57		
	Water body			
	presence vs absence	-0.61 (0.42), 0.15	-135.0, 270.0 (308)	286.0
*L*. *borgpetersenii*	Water body			
	presence vs absence	-1.08 (0.50), **0.03**		
	Trap location			
	scrub *vs* forest	16.95 (2498.27), 0.99		
	garden/yard *vs* forest	17.45 (2498.27), 0.99		
	domestic dwelling *vs* forest	16.85 (2498.27), 0.99		
	sewer *vs* forest	17.65 (2498.27), 0.99	-100.7, 201.4 (309)	215.4

Values are shown for i) the estimate of the logit function (estimate) with standard deviation (SD) and p-value (P), (ii) the log likelihood with residual deviance and degrees of freedom (DF) and (iii) the corrected Akaike information criteria (AICc) of the best selected model.

The built environment GLM further investigated the effects of building type, building condition, trap location and site location on rodent infection with *Leptospira* (S5 Appendix). The first top model demonstrated that individual rodents trapped in or near buildings with mixed commercial and residential uses were more likely to be infected by both *Leptospira* species (P = 0.001, Table 3). Moreover, the presence of institutional buildings within a site (e.g., churches, schools, etc.) also appeared to increase the risk of rodent infection by *L*. *interrogans* (P = 0.038, Table 3).

**Table 3:** Results of the first best built environment GLM (with logit link function) explaining the occurrence of (1) all *Leptospira*, (2) *L*. *interrogans* and (3) *L*. *borgpetersenii* in rodents as a function of site location, trap location, building type and building condition (S5 Appendix).

	Explanatory variables	Estimate (SD), P	Log likelihood, dev (DF)	AICc
All *Leptospira*	Building type			
	commercial/residential *vs* residential	1.77 (0.34), **0.0001**		
	institutional *vs* residential	0.52 (0.72), 0.47	-158.5, 352.3 (270)	325.0
*L*. *interrogans*	Building type			
	commercial/residential *vs* residential	2.16 (0.54), **0.0001**		
	institutional *vs* residential	1.80 (0.83), **0.029**	-121.1, 267.9 (270)	248.2
*L*. *borgpetersenii*	Building type			
	commercial/residential *vs* residential	1.05 (0.44), **0.017**		
	institutional *vs* residential	-15.1 (1057.3), 0.99	-104.3, 219.7 (270)	214.7

Values are shown for i) the estimate of the logit function (estimate) with standard deviation (SD) and p-value (P), (ii) the log likelihood with residual deviance (dev) and degrees of freedom (DF) and (iii) the corrected Akaike information criteria (AICc) of the best selected model.

## Discussion

The results of this study reveal that the prevalence of *Leptospira* spp. in rodents is influenced by a number of environmental factors, and that these may vary depending on the species of *Leptospira* considered. Overall, *Leptospira* prevalence increased with increasing anthropogenic influence across the landscape, with a significantly higher proportion of infected rodents observed at the urban location. In particular, *L*. *borgpetersenii* was most commonly found at sites without a natural body of water, whilst *L*. *interrogans* infection was most prevalent among rodents inhabiting sites with low forest cover. For sites within the built environment, the type of buildings present was also found to have an impact on the prevalence of both *Leptospira* species.

The overall prevalence of pathogenic *Leptospira* spp. in rodents in this study was slightly higher (32%) than those observed in other studies from the Southeast Asian region (6–27%), and was also higher than previously identified from rodents trapped in urban areas of Sarawak (5.6%, N = 107 rodents) [6,22,42–46]. While the discrepancy between our results and those of Pui et al. [22] are not straightforward to explain, they may be due to differences in sampling sites, organs tested and laboratory methodology. Pui et al., cultured all samples prior to detection with a single PCR assay, unlike the direct-detection methodology with multiple primer sets applied in the current study. Although the approach of Pui et al. is common, growing *Leptospira* in vitro is well-known to be challenging and can be biased by species and serovar, which may result in a lower reported prevalence.

Rural habitats have repeatedly been associated with an increased risk of leptospirosis due to associations with some types of agriculture (e.g. rice farming) and outdoor recreational activities [47,48]. As a result, the majority of research on the ecology and distribution of *Leptospira* spp. in rodents has been performed in rural environments, and the ecological drivers and risk factors for zoonotic infection in these habitats are relatively well documented. In contrast, *Leptospira* ecology in urban environments has received considerably less attention, despite the abundance of rodents and other potential hosts in urban environments, and clear evidence of human infection [49–52]. In addition, and as observed in this study, most surveys of urban rats have found a high prevalence of pathogenic *Leptospira* spp. [42,53–56]. The high population densities that rodents can reach in urban areas and the resulting frequency of human-rodent contact suggests that a real risk of human infection is present, even when infection prevalence in rodents is low [57,58]. From the results of this study we are unable to determine if the *Leptospira* spp. carried by rodents in and around Kuching are associated with human infection, or what the relevant risk factors for zoonotic transmission may be. However, previous work has assessed serovar diversity in both soil and rodents in urban Sarawak, and predominantly identified *L*. *interrogans* serovar Icterohaemorrhagiae [22]. This serovar is commonly associated with rodents and has been linked to human disease in Sarawak and other regions of Malaysia [59–62]. In addition, serovar Sarawak (Lepto 175) has also been detected in both humans and rodents in Sarawak but has not yet been confirmed as an agent of human disease [25,62,63]. Taken together this suggests that rodents, including the species sampled in this study, are likely a source of human infection in Sarawak and the Southeast Asian region.

In recent years, several leptospirosis outbreaks in Malaysia have been linked to outdoor activities (e.g. hiking, water-sports) in natural environments [23,42]. Reflective of this risk, both species of *Leptospira* were detected in vegetated areas across the landscape in this study. Infected rodents were detected in disturbed forests, recreational parks and vacant lots, all of which are utilized to varying degrees by people, and which provide additional interfaces for human exposure to *Leptospira*. Across our study sites, city parks are used extensively for sporting and social activities, vacant lots for edible plant foraging and small-scale fruit and vegetable cultivation (personal observation), and disturbed forests for farming, hunting and foraging, as well as recreational activities [64,65].

In contrast, we identified an unexpected association between the presence of *L*. *interrogans* and reduced forest cover at a site, which may indicate that transmission is favored in more cleared (and disturbed) habitats [6,66]. However, this trend may be related to the ecology of the dominant rodent species assessed in this study. Across Southeast Asia, both members of the *R*. *rattus* super-group and *S*. *muelleri* are often found at higher abundance in disturbed and urban habitats compared to more pristine, forested habitats [67,68]. It is therefore possible that rodent population density, which was not measured here but did appear low in forested areas, is a factor that inhibits *Leptospira* spp. transmission. Alternatively, the lower infection prevalence observed at sites characterized by high forest cover may simply be a result of the relatively small number of rodents trapped at these sites. Rodent abundance may also be related to the association we identified between the presence of buildings with mixed commercial and residential uses, and an increased prevalence of both *Leptospira* investigated here. This type of building, which often has a shop, restaurant or market on the ground floor and higher-density accommodation above, is the primary building type found in the center of Kuching and at focal points of human activity in its suburbs. The disposal of waste from these premises is often informal and directed towards the sewerage system, providing an ample source of food for rodents. As sewers also provide access to water and shelter from most predators, they are regularly favored by urban rodents such as *R*. *norvegicus*, which can become extremely abundant in urban environments [55]. The high rodent population densities that can occur in such settings may promote the circulation of *Leptospira* and increase the risk of zoonotic transmission in urban environments. Indeed, living close to open sewers has been identified as a risk factor for human *Leptospira* infection in Salvador, Brazil, and occupational risks have been identified for town cleaners and sewage workers in other cities [24,69–71]. The drivers behind the association between *L*. *interrogans* positive rodents and sites with institutional buildings is less clear, although this may be an artifact of our analysis as there were only four sites in this category. However, it is worth noting that as none of these sites were rural, this association may also be reflective of benefits related to higher levels of urbanization.

It is surprising that the presence of *L*. *borgpetersenii* at a site was significantly more likely if natural bodies of water were absent, and may reflect the evolution of this species towards direct transmission between hosts [5]. This is reflected in the findings of another Southeast Asian study, which found *L*. *borgpetersenii* to be abundant in both dry and humid habitats, with the highest prevalence in non-floodable lands such as orchards, plantations and shrubby wasteland [6]. However, it may be worth noting that although sewers were accounted for by the variable ‘trap location’ in our analyses, they were not part of the variable ‘water body’ due to their artificial nature and the inconsistent presence of water in this environment. As approximately half of all *Leptospira*-positive rodents (51/100) were trapped in sewers, their exclusion from this category may have influenced these results. In addition, ‘trap location’ was not selected in the best top model for *L*. *borgpetersenii* (Table 2), but it was selected in the second and third top models, indicating that it may have some influence on *L*. *borgpetersenii* infection prevalence (S4 Appendix).

This study focused on identifying environmental factors that influence the prevalence of *Leptospira* spp. in urbanizing environments; however, the environment can both directly and indirectly influence the circulation of *Leptospira* (i.e. by shaping host ecology). While we are unable to distinguish between these two modes of action in this study, our choice to explore the ecology of *Leptospira* across all rodents collectively is supported by several factors: 1) *Leptospira* are host generalists; 2) rodent species are not known to differ in competence; 3) a similar ecological study of *Leptospira* in Thailand found no impact of rodent species [6]; 4) individuals from *R*. *rattus* R3 and *S*. *muelleri* (comprising 84% of all captures in this study) were both found in urban, developing and rural locations, including at some of the same sites. However, infection prevalence did vary between these two species, with 42.5% of *R*. *rattus* R3 and 23.0% of *S*. *muelleri* individuals infected, suggesting that either the former is more likely to become infected, or that this species prefers to inhabit environments that promote the circulation of *Leptospira*. For example, *R*. *rattus* R3 was commonly caught in sewers in our study (73/165 captures), and large numbers of animals were often observed at these sites, suggestive of high population density. These conditions may promote the circulation of *Leptospira*, particularly for species such as *L*. *borgpetersenii*, which rely on direct transmission between hosts [5].

The number of reported cases of leptospirosis in Malaysia has increased considerably in recent years [23]. Although many cases are still documented in rural areas, zoonotic transmission is also clearly a feature of urban living, with some occupations (i.e. garbage collectors, town cleaners) associated with a higher risk of infection [24,69]. The high prevalence of *Leptospira* observed here and the importance of rodents as sources for human disease, suggests that the ecology and dynamics of rodent-associated transmission in urban Kuching warrants further study and may be required to prevent ongoing human disease. With the increasing loss of natural habitats and continuing urbanization occurring across the globe, the majority of zoonotic transmission events are anticipated to occur in urban settings. It is therefore essential to develop a thorough understanding of the drivers of pathogen transmission and zoonotic infection that occur in the ecologically and demographically complex urban environment.

## Supporting information

S1 Appendix Leptospira DNA sequences. Sequences too short to submit to GenBank are included in FASTA format. The sample ID and PCR assay which yielded each sequence (rpoB, flaB or 16S) is indicated in each sequence name.
(FASTA)

S2 Appendix Differences in the prevalence of *Leptospira* in rodents between urban, developing and rural site locations. (PDF)

S3 Appendix Matrix of Goodman and Kruskal’s τ among categorical variables describing the environments. The bottom left diagonal shows τ measurements of the predictive effect of each row variable on each column variable, and the top right diagonal shows τ measurements of the reverse association. For example, “forest cover” has a weak predictive effect on “building type” (τ = 0.37), but the reverse association is much stronger (τ = 0.50).
(PDF)

S4 Appendix Comparison of the 3 top models from the global GLMM. Variables included site location, trap location, forest cover, dominant land-cover type and waterbody on individual rodent infection by (1) all types of *Leptospira*, (2) *L*. *interrogans* and (3) *L*. *borgpetersenii*. Models are ranked from lowest to highest support according AICc. *K* is the number of estimated parameters, AICc the selection criterion, and w_r_ the Akaike weights.
(PDF)

S5 Appendix Comparison of the 3 top models from the built environment GLM. Variables included site location, trap location, building type and building condition on individual rodent infection by (1) all types of *Leptospira*, (2) *L*. *interrogans* and (3) *L*. *borgpetersenii*. Models are ranked from lowest to highest support according to AICc. *K* is the number of estimated parameters, AICc the selection criterion, and w_r_ the Akaike weights.
(PDF)
