# Supplementary material for: Association of rodent-borne Leptospira spp. with urban environments in Malaysian Borneo
Source: PLoS Negl Trop Dis. 2019 Feb 27;13(2):e0007141. doi: 10.1371/journal.pntd.0007141 (PMC6411199; doi:10.1371/journal.pntd.0007141)
Supplement: S2 Appendix — (PDF) [file pntd.0007141.s002.pdf]

S2 Appendix. Differences in the prevalence of *Leptospira* in rodents between urban, developing and rural site locations, as tested using Chi Squared tests.

	**Chi-squared (χ^2) value (p value)**		
	All *Leptospira* spp.	*L. borgpetersenii*	*L. interrogans*
**All locations**	23.903 ($p < 0.01$)	7.148 ($p < 0.05$)	11.790 ($p < 0.01$)
**Urban vs Developing**	4.164 ($p < 0.05$)	3.769 ($p > 0.05$)	0.505 ($p > 0.05$)
**Urban vs Rural**	23.976 ($p < 0.01$)	10.945 ($p < 0.01$)	7.287 ($p < 0.01$)
**Developing vs Rural**	10.886 ($p < 0.01$)	3.185 ($p > 0.05$)	4.754 ($p < 0.05$)
