# Supplementary material for: Association of rodent-borne Leptospira spp. with urban environments in Malaysian Borneo
Source: PLoS Negl Trop Dis. 2019 Feb 27;13(2):e0007141. doi: 10.1371/journal.pntd.0007141 (PMC6411199; doi:10.1371/journal.pntd.0007141)
Supplement: S3 Appendix — The bottom left diagonal shows τ measurements of the predictive effect of each row variable on each column variable, and the top right diagonal shows τ measurements of the reverse association. For example, “forest cover” has a weak predictive effect on “building type” (τ = 0.37), but the reverse association is much stronger (τ = 0.50). (PDF) [file pntd.0007141.s003.pdf]

S3 Appendix. **Matrix of Goodman and Kruskal's τ among categorical variables describing environment**. The bottom left diagonal shows τ measurements of the predictive effect of each row variable on each column variable, and the top right diagonal shows τ measurements of the reverse association. For example, "forest cover" has weak predictive effect on "building type" (τ= 0.37), but the reverse association is much stronger (τ= 0.50).

	site location	trap location	dominant land-cover type	forest cover	water body	building type	building condition	rodent species
site location	K = 3	0.14	0.09	0.29	0.17	0.27	0.09	0.03
trap location	0.22	K = 5	0.55	0.38	0.35	0.37	0.17	0.32
dominant land-cover type	0.13	0.57	K = 4	0.44	0.39	0.4	0.15	0.38
forest cover	0.2	0.19	0.28	K = 3	0.39	0.37	0.17	0.23
water body	0.07	0.14	0.16	0.27	K = 2	0.19	0.08	0.18
building type	0.29	0.29	0.37	0.5	0.38	K = 5	0.41	0.27
building condition	0.14	0.18	0.19	0.29	0.19	0.38	K = 5	0.16
rodent species	0.12	0.34	0.3	0.37	0.31	0.32	0.22	K = 9
