# Supplementary material for: Association of rodent-borne Leptospira spp. with urban environments in Malaysian Borneo
Source: PLoS Negl Trop Dis. 2019 Feb 27;13(2):e0007141. doi: 10.1371/journal.pntd.0007141 (PMC6411199; doi:10.1371/journal.pntd.0007141)
Supplement: S4 Appendix — Variables included site location, trap location, forest cover, dominant land-cover type and waterbody on individual rodent infection by (1) all types of Leptospira, (2) L. interrogans and (3) L. borgpetersenii. Models are ranked from lowest to highest support according AICc. K is the number of estimated parameters, AICc the selection criterion, and wr the Akaike weights. (PDF) [file pntd.0007141.s004.pdf]

S4 Appendix. Comparison of the 3 top models from the global GLMM. Variables included site location, trap location, forest cover, dominant land-cover type, and waterbody on individual rodent infection by (1) all types of *Leptospira,* (2) *L. interrogans* and (3) *L. borgpetersenii.* Models are ranked from lowest to highest support according AICc. *K* is the number of estimated parameters, AICc the selection criterion, and w_r the Akaike weights.

Leptospira	**Models (best top 3)**	***K***	**AICc**	**w_r**
All *Leptospira*	Forest cover + water body	3	354.5	0.271
	Forest cover + dominant land-cover type + water body	4	355.9	0.132
	Forest cover + dominant land-cover type + water body + trap location	5	355.9	0.132
L. interrogans	Forest cover + dominant land-cover type + water body	4	286.4	0.130
	Forest cover + dominant land-cover type + water body + trap location	5	286.4	0.130
	Forest cover + dominant land-cover type	3	286.5	0.128
L. borgpetersonni	Water body + trap location	3	215.7	0.226
	dominant land-cover type + water body	3	217.2	0.110
	Forest cover + water body	3	217.8	0.078
