# Supplementary material for: Association of rodent-borne Leptospira spp. with urban environments in Malaysian Borneo
Source: PLoS Negl Trop Dis. 2019 Feb 27;13(2):e0007141. doi: 10.1371/journal.pntd.0007141 (PMC6411199; doi:10.1371/journal.pntd.0007141)
Supplement: S5 Appendix — Variables included site location, trap location, building type and building condition on individual rodent infection by (1) all types of Leptospira, (2) L. interrogans and (3) L. borgpetersenii. Models are ranked from lowest to highest support according to AICc. K is the number of estimated parameters, AICc the selection criterion, and wr the Akaike weights. (PDF) [file pntd.0007141.s005.pdf]

S5 Appendix. Comparison of the 3 top models from the built environment GLM. Variables included site location, trap location, building type and building condition on individual rodent infection by (1) all types of *Leptospira,* (2) *L. interrogans* and (3) *L. borgpetersenii*. Models are ranked from lowest to highest support according to AICc. *K* is the number of estimated parameters, AICc the selection criterion, and w_r the Akaike weights.

Leptospira	**Models (best top 3)**	***K***	**AICc**	$\mathbf{w_r}$
All *Leptospira*	Building type	2	323.1	0.295
	Site location + building type	3	323.9	0.201
	Site location + building condition	3	325.3	0.102
L. interrogans	Building type	2	248.3	0.513
	Building type + building condition	3	251.3	0.113
	Site location + building type	3	251.7	0.090
L. borgpetersonni	Building type	2	214.8	0.308
	Trap location	2	215.1	0.263
	Trap location + building condition	3	217.2	0.090
